# Investigation of the Vitamin D Receptor Polymorphisms in Acromegaly Patients

**DOI:** 10.1155/2015/625981

**Published:** 2015-03-08

**Authors:** Muzaffer Ilhan, Bahar Toptas-Hekimoglu, Ilhan Yaylim, Seda Turgut, Saime Turan, Ozcan Karaman, Ertugrul Tasan

**Affiliations:** ^1^Department of Endocrinology and Metabolism, Bezmialem University, 34093 Istanbul, Turkey; ^2^Department of Molecular Medicine, The Institute of Experimental Medicine, 34093 Istanbul, Turkey; ^3^Department of Internal Medicine, Bezmialem University, 34093 Istanbul, Turkey

## Abstract

*Objective*. The genetic structural alterations in the majority of somatotroph adenomas are not clarified and the search for novel candidate genes is still a challenge. We aimed to investigate possible associations between vitamin D receptor (VDR) polymorphisms and acromegaly. *Design, Patients, and Methods*. 52 acromegaly patients (mean age 45.7 ± 1.9 years) and 83 controls (mean age 43.1 ± 2.6 years) were recruited to the study. VDR polymorphism was determined by polymerase chain reaction-based restriction fragment length polymorphism methods. *Results*. The distribution of VDR genotypes showed a significant difference in the frequencies of VDR FokI genotypes between patients and controls (*P* = 0.034). VDR FokI ff genotype was significantly decreased in acromegaly patients (*P* = 0.035) and carriers of FokI Ff genotype had a 1.5-fold increased risk for acromegaly (OR: 1.5, 95% CI: 1.07–2.1; *P* = 0.020). IGF1 levels after treatment were significantly higher in patients carrying the Ff genotype compared to carrying ff genotype (*P* = 0.0049). 25(OH)D3 levels were significantly lower in acromegaly patients (*P* < 0.001). *Conclusions*. Our study suggests that VDR FokI genotypes might affect the development of acromegaly and VDR polymorphisms may play a role in the course of acromegaly as a consequence of altering hormonal status.

## 1. Introduction

Acromegaly is a rare disease characterized with elevated levels of growth hormone (GH) and insulin like growth factor 1 (IGF1) [1]. Acromegaly disease is almost always caused by pituitary GH overexpressing adenomas [2]. A few germline mutations have been shown to convey an inherited risk of somatotroph adenomas; however, the vast majority of these tumors are sporadic origin [3]. To date, the most frequent somatic molecular alterations identified are activating mutations in the guanine nucleotide-binding a-subunit 1 gene (GNAS) [4]. These mutations induce adenylyl cyclase activation, cellular proliferation, and GH oversecretion. However, no Gs alpha subunit (Gs*α*) mutations have been found in about two-thirds of sporadic acromegaly tumors indicating that there are other unknown mechanisms. Other molecular alterations have been reported, such as cAMP response element-binding protein (CREB) activation and pituitary tumor-transforming gene (PTTG) overexpression [5, 6]; however, the genetic structural alterations in the majority of sporadic somatotroph adenomas remain unknown and the seeking for novel candidate genes is still a challenge.

Beyond calcium homeostasis, vitamin D is a well-known potent regulator of cell growth and differentiation [7]. 1,25-Dihydroxyvitamin D3 [1,25(OH)2D3, calcitriol] binds to a corresponding intranuclear receptor and interacts with various cell cycle regulators identified in numerous genes involved in cellular growth, differentiation, apoptosis, and invasion by tumor cells such as the human p21/WAF1, cyclin A and cyclin E, human c-fos and c-myc, and the human retinoblastoma gene [8]. The vitamin D receptor (VDR) is encoded by a large gene located on chromosome 12q13-14 and forms a heterodimer with retinoid X receptors (RXRs) and both VDR and RXRs are the members of the steroid nuclear receptor superfamily [9]. RXRs binding is essential for transcriptional activation of VDR [10]. While the function of the RXRs in tumorigenesis has not been studied as thoroughly as that of VDR, RXRs have been implicated in the tumorigenesis of somatotroph adenomas [11].

There are more than 470 single nucleotide polymorphisms (SNPs) identified in the VDR gene and locations of the polymorphisms determine their functional roles [12, 13]. The polymorphisms most extensively focused on are rs10735810 or FokI in exon 2, rs1544410 or BsmI in intron 8, rs731236 or TaqI in exon 9, rs7975232 or ApaI in intron 8, rs757343 or Tru91 in intron 8, and the poly(A) mononucleotide repeat in the 3′-UTR [12]. TaqI and ApaI polymorphisms are considered to be silent nucleotide polymorphisms. However, the VDR FokI polymorphism leads to T > C substitution (T ≡ F, C ≡ f allele), resulting in a less potent transcriptional activator product [14]. On the other hand, the F allele leads to higher transcriptional and functional activities [13].

In the present study we aimed to investigate possible associations of vitamin D receptor polymorphisms and VDR activity levels with acromegaly and acromegaly disease characteristics.

## 2. Material and Methods

### 2.1. Patients and Hormone Assays

52 acromegaly patients (31 females and 21 males) and 83 healthy controls (53 females and 30 males) were recruited to the study between 2011 and 2013 in Bezmialem University Hospital Endocrinology Clinic, Turkey. Our patients and controls were selected from the Turkish population. The diagnosis of acromegaly disease was based on clinical features and was confirmed by GH levels <0.4 ng/mL after an oral glucose tolerance test or high age-matched IGF1 levels [15]. Magnetic resonance imaging (MRI) of the hypophysis was performed on all acromegalic patients and maximum diameter was determined as the tumor size. Acromegaly disease is considered to be in biochemically remission, if nadir GH < 0.4 ng/mL during an OGTT or under random GH < 1 ng/mL for patients receiving medical treatment and normal age matched IGF1 levels [15]. IGF1 values were adjusted as IGF1/upper limit of normal range (ULN)∗100.

Blood GH and IGF1 levels were assayed using a chemiluminescence immunometric assay (Siemens Advia-Centaur USA). Age-related reference ranges for IGF1 were as follows: 18–20 y: 197–956; 20–23 y: 215–628; 23–25 y: 169–591; 25–30 y: 119–476; 30–40 y: 100–494; 40–50 y: 101–303; >50 y: 78–258 (ng∖mL).

For the measurement of 25-hydroxyvitamin D3 [25(OH)D3] levels, blood was collected in polystyrene tubes and centrifuged immediately. The serum was stored at −30°C until 25(OH)D3 levels were determined by enzyme-linked immunoassay (Catalog number K2110; Immundiagnostik AG, Stubenwald-Alee 8a, D64625 Bensheim Austria). Reference range for 25(OH)D3 was 6.4–24 nmol/L.

This study was approved by the local ethics committee of Bezmialem Vakif University and informed consent was obtained in all cases.

### 2.2. VDR Genotyping Methods

Blood specimens were collected in tubes containing EDTA, and DNA samples were extracted from whole blood by a salting out procedure [16]. Genotyping was performed by the polymerase chain reaction (PCR) and restriction fragment length polymorphism (RF LP). For VDR gene TaqI and ApaI polymorphisms, sense primer was 5′-C AG AGC ATG GAC AGG GAG CAA G-3′ and antisense primer was 5′-GCA ACT CCT CAT GGG C TG AGG TCT CA-3′ [17]. For detection of the TaqI and ApaI RFLP, 50–100 ng genomic DNA was amplified with 1x polymerase chain reaction buffer, 3 mM MgCl2, 0.2 mM of each dNTP, 0.2 mM of each primer, and Taq polymerase in a 50 *μ*L reaction volume. Samples were denaturated at 94°C and passed through 5 cycles of 94°C for 45 sec, 64°C for 60 sec, and 72°C for 2 min and a further 25 cycles of 94°C for 30 sec, 64°C for 30 sec, and 72°C for 45 sec. PCR products were digested with TaqI and ApaI restriction enzyme at 65°C and 37°C electrophoresed on 2% agarose gels and stained with ethidium bromide. TaqI polymorphism genotypes were determined as TT (490 and 245 bp), Tt (490, 290, 245, and 205 bp), or tt (290, 245, and 205 bp) [17] and ApaI polymorphism genotypes were determined as AA (740 bp), Aa (740, 530, and 210 bp), or aa (530 and 210 bp) [18]. The primers (MBI Fermentas, Lithuania) for FokI polymorphism were 5′-GAT GCC AGC TGG CCC TGG CAC TG-3′ and 5′-ATG GAA ACA CCT TGC TTC TTC TCC CTC-3′. The DNA template was amplified by PCR using 3 mM MgCl2, 0.2 mM of each dNTP, 0.25 mM of each primer, and Taq polymerase (MBI Fermentas, Lithuania) in a 50 *μ*L final volume. The PCR conditions involved an initial denaturation of 4 min at 94°C, followed by 30 cycles of 94°C for 1 min, annealing at 60°C for 1 min, and extension at 72°C for 1 min. A final extension step at 72°C for 4 min was also studied. PCR products were digested with FokI restriction enzyme (MBI Fermentas, Lithuania) at 37°C for 3 h followed by electrophoresis in a 2% agarose gel. The FF genotype (homozygote of common allele) shows only one band of 272 bp in agarose gel. The ff genotype (homozygote of infrequent allele) generates two fragments of 198 and 74 bp. The heterozygote displays three fragments (272, 198, and 74 bp) [17].

### 2.3. Statistical Analysis

Differences in the frequencies of the VDR polymorphisms between acromegaly patients and the control group were analyzed using the Chi-square test. Hardy Weinberg equilibrium (HWE) was tested for all polymorphisms. The associations in means between groups were analyzed by Student's *t*-test or ANOVA and Mann-Whitney *U* or Kruskal-Wallis tests depending on data distribution. Spearman's coefficient was applied to test for bivariate correlations. The relative associations between acromegaly patients and controls were assessed by calculating crude Gart's odds ratios (ORs) and 95% confidence intervals (95% CIs). The threshold for significance was *P* < 0.05. The SPSS version 11.0 for Windows was used to perform statistical analysis (SPSS Inc. Chicago, IL, USA). Haplotype frequencies, *D*′ (a normalized measure of linkage disequilibrium between the two loci), and *r* ^2^ (the correlation coefficient between the two loci) were calculated using Haploview software version 4.2 [19].

## 3. Results

Clinical characteristics of the patients with acromegaly and control group were summarized in Table 1. There was no significant difference in gender and age between patients and healthy subjects (*P* > 0.05). Genotype distributions for VDR ApaI and TaqI polymorphisms, both in control and patient group, and for FokI polymorphisms in the control group did not deviate significantly from HWE (*P* > 0.05). However, FokI genotype distributions for the patient group did deviate significantly from HWE (*P* = 0.04 for patients).

The mean 25(OH)D3 level in acromegaly patients (5.5 ± 1.1 nmol∖L) was significantly lower than in controls (29.9 ± 2.1 nmol∖L) (*P* < 0.001). No association was found between 25(OH)D3 levels and any disease characteristics such as tumor size or remission status. Also, 25(OH)D3 levels were not correlated with hormone levels such as GH, IGF1, or IGF1 (% ULN). While GH levels before treatment were not correlated with IGF1 levels before treatment, GH levels after treatment were correlated with IGF1 levels after treatment (*P* = 0.003, *r* = 0.44).

We examined three genetic variants of the VDR in acromegaly patients. The distribution of acromegaly patients and controls associated with VDR genotypes is shown in Table 2. There was a significant difference in the frequencies of VDR FokI genotypes between patients and control groups (*P* = 0.034). VDR FokI ff genotype was significantly decreased in acromegaly patients (9.6%) compared to controls (24.1%) (OR: 0.4, 95% CI: 0.16–0.99; *P* = 0.035). Carriers of FokI Ff genotype had a 1.5-fold increased risk for acromegaly disease (OR: 1.5, 95% CI: 1.07–2.1; *P* = 0.020). There was no significant association of either TaqI or ApaI genotypes with acromegaly patients and controls (*P* > 0.05).

Vitamin D haplotypes (TaqI, FokI, and ApaI) were evaluated for association with acromegaly (data not shown). Haplotype analysis revealed that there was no relationship between VDR haplotypes and acromegaly cases (for acromegaly patients *D*′ 0.24 LOD: 0.47 *r*-squared 0.022).

We also analyzed the association of these three polymorphisms with characteristics of acromegaly (Table 3). There was no significant difference in either pretreatment GH levels or serum vitamin D levels across TaqI TT, Tt, and tt genotypes taken as a whole (*P* > 0.05 for both).

Another finding of this study was the relationship between FokI genotype and hormonal status of acromegaly patients after treatment. We observed that patients with the FF genotype had higher GH levels after treatment than patients with ff genotype, but the difference was only marginally significant (*P* = 0.051). In addition IGF1 levels after treatment were significantly higher in patients carrying the Ff genotype compared to carrying ff genotype (*P* = 0.0049). No other associations were found between FokI, ApaI, or TaqI genotypes and disease characteristics.

## 4. Discussion

Vitamin D plays a crucial role in a broad variety of hormonal regulations including bone metabolism, immune response, cell proliferation, and differentiation [20]. Many common diseases such as rickets, diabetes, cardiovascular diseases, autoimmune diseases, and cancers have been associated with vitamin D deficiency [21]. Excessive pituitary somatotroph cell proliferation and unrestrained GH hypersecretion are the main pathogenetic events in the course of acromegaly disease development. GH secreting adenomas of anterior pituitary gland which may arise from clonal expansion of somatotroph cells almost always underlie acromegaly disease and this monoclonal origin suggests that intrinsic genetic alterations can play an important role for possible tumorigenic initiating mechanisms [22]. Previous studies indicate that 1,25(OH)2D3, the most active form of vitamin D, regulates the growth and differentiation of various cell types [23]. Moreover, there is growing evidence of regulatory effects on cell death and tumor invasion on tumorigenesis [24]. The VDR gene is one of the most widely studied tumorigenesis-related genes and occurrence of many different types of tumors has been associated with VDR polymorphisms. To our knowledge, this is the first study investigating VDR polymorphisms in acromegaly disease.

In this study we found that, while the VDR FokI ff genotype was associated with a decreased risk, FokI Ff genotype was associated with a significantly increased risk of acromegaly. The FokI polymorphism (thymine/cytosine polymorphism) is the only known VDR gene polymorphism that leads to the generation of an altered protein [25]. The f allele (thymine variant) results in the generation of a longer VDR protein and less transcriptional activity; the F allele results in 1.7-fold more transcriptional activity [26]. Although the majority of the physiological effects of VDR polymorphisms are not fully understood, it has been postulated that these polymorphisms may influence the risk of cancer occurrence [27]. In previous studies VDR polymorphisms have been associated with the risk of breast, prostate, skin, ovary, and bladder carcinoma [28–31] On the other hand, other studies have found no significant association between VDR polymorphisms and cancers [32]. In our study, VDR haplotypes showed no significant difference between patient and control groups. However, it can be noted that FokI polymorphism has not been reported to be in linkage disequilibrium with other VDR polymorphisms and has been considered to be an independent risk marker [33].

In this study, we observed that IGF1 levels after treatment were significantly higher in patients carrying Ff genotype compared to these carrying the ff genotype. However, there was no significant difference across FokI genotypes when the IGF levels after treatment were adjusted according to the upper limit of normal IGF1. The VDR TaqI polymorphism is located in a regulatory area rather than in a coding exon and considered to be silent single nucleotide polymorphisms [34]. Although these polymorphisms do not alter the amino acid sequence of the encoded protein, they can affect gene expression through regulation of mRNA stability [35].

Several studies have indicated that acromegaly can be associated with increased 1,25(OH)2D3 levels in treatment naive patients [36]. Consistent with Takamoto et al., we found significantly decreased 25(OH)D3 levels in the patient group without any vitamin D supplement [37]. It is suggested that IGF1 stimulation can overactivate renal 1*α*-hydroxylase and increase 1,25(OH)2D3 production [38]. Thereby, decreased 25(OH)D3 level can be an expected finding in acromegaly patients. Along with the evidences of the high occurrence of vertebral fractures despite normal bone mineral density in acromegaly [39], vitamin D status must be considered during follow-up of these patients.

Limitation of the present study was that measurement of exogenous environmental factors effecting vitamin D levels such as UV exposure or daily nutrition containing vitamin D was lacking. Secondly the sample size in the acromegaly group was relatively small due to the rarity of acromegaly and the findings of this study should be demonstrated in larger samples. This study was also a single center based case-control study which could be unrepresentative of acromegaly patients in general population. However, we found similar results compared to previous Turkish studies for the distribution of VDR genotypes [40]. Also it should be noted that our hospital is a reference center admitting patients from all over the country.

In conclusion these findings suggest a possible role of VDR FokI polymorphism in the risk of acromegaly. Vitamin D levels should be considered and followed up in the treatment of acromegaly patients. VDR genotypes can play a role as a consequence of altering hormonal status in the course of acromegaly. Further studies with large numbers are needed to clarify these findings.

## Figures and Tables

**Table 1:** Demographic characteristics of acromegaly patients and control group.

	Acromegaly (*n* = 52)	Control (*n* = 83)
Gender (*n*, %)		
Female	31 (59.6)	53 (63.9)
Male	21 (40.4)	30 (36.1)
Age (y)	45.7 ± 1.9	43.1 ± 2.6
Age onset (y)	39.5 ± 1.9	
Tumor size before treatment (*n*, %)		
Macroadenoma	36 (81.8)	
Microadenoma	8 (18.2)	
Number of resection (*n*, %)		
0	8 (17.8)	
1	29 (64.4)	
≥2	8 (17.8)	
IGF1 (ng/mL)		
Before treatment	882.5 ± 56	
After treatment	234.6 ± 21.3	
IGF1 (% ULN)		
Before treatment	330.0 ± 21	
After treatment	100.8 ± 10.4	
GH (ng/mL)		
Before treatment	18.3 ± 2.9	
After treatment	1.3 ± 0.2	
Remission status (*n*, %)		
Controlled	36 (80)	
Uncontrolled	9 (20)	
Tumor size after treatment (*n*, %)		
≥1 cm	10 (22.7)	
<1 cm	34 (77.3)	
25(OH)D3 (nmol/L)^*^	5.5 ± 1.1	29.9 ± 2.1

Data are shown as mean ± standard error. IGF1 (%ULN): IGF1/upper limit of normal (age-matched) ∗ 100.

^*^ *P* < 0.001.

**Table 2:** Distribution of vitamin D receptor (VDR) genotypes and alleles in acromegaly patients and control subjects.

	Patients (*n*, %)	Controls (*n*, %)
TaqI genotypes/alleles		
TT	19 (36.5)	38 (45.8)
Tt	29 (55.8)	38 (45.8)
tt	4 (7.7)	7 (8.4)
T	67 (64.4)	114 (68.7)
t	37 (35.6)	52 (31.3)
ApaI genotypes/alleles		
AA	20 (38.5)	28 (33.7)
Aa	25 (48.1)	43 (51.8)
aa	7 (13.5)	12 (14.5)
A	65 (62.5)	99 (59.6)
a	39 (37.5)	67 (40.4)
FokI genotypes^a^/alleles		
FF	15 (28.8)	29 (34.9)
Ff^b^	32 (61.5)	34 (41)
ff^c^	5 (9.6)	20 (24.1)
F	62 (59.6)	92 (55.4)
f	42 (40.4)	74 (44.6)

^a^Frequencies of FokI genotypes between patients and controls (*P* = 0.034).

^b^Ff versus FF + ff (OR: 1.5, 95% CI: 1.07–2.1; *P* = 0.020).

^c^ff versus FF + Ff (OR: 0.4, 95% CI: 0.16–0.99; *P* = 0.035).

**Table 3:** The comparisons of characteristics among the VDR genotypes in acromegaly patients.

Genotypes	Age onset (years)	GH (ng/mL)		IGF1 (ng/mL)		IGF1 (% ULN)		Tumor size before treatment (*n*, %)		Remission (*n*, %)		25(OH)D3 (nmol/L)
		Before treatment	After treatment	Before treatment	After treatment	Before treatment	After treatment	<1 cm	≥1 cm	Controlled	Uncontrolled	
ApaI												
AA	39.5 ± 2.6	21.4 ± 5	1.15 ± 0.3	885.4 ± 78.6	248.4 ± 30	321.7 ± 30.6	103.5 ± 12.3	3 (37.5)	14 (38.9)	12 (33.3)	3 (33.3)	6.5 ± 2.5
Aa	40.4 ± 3.2	18 ± 4.3	1.1 ± 0.2	896.4 ± 100.7	228 ± 33.2	335.7 ± 33.6	101.7 ± 18.4	4 (50)	18 (50)	19 (52.8)	4 (44.4)	3.4 ± 0.9
aa	36.1 ± 5.5	11.1 ± 4.1	2.1 ± 1	829.6 ± 89.2	226.4 ± 59.3	335.4 ± 63.6	92.1 ± 18.6	1 (12.5)	4 (11.1)	5 (13.9)	2 (22.2)	10 ± 2.2
FokI												
FF	39.7 ± 3	12.5 ± 4.1	1.6 ± 0.3	907.5 ± 93.1	249.5 ± 39.9	344.2 ± 36.8	106.8 ± 20.1	3 (37.5)	10 (27.8)	9 (25)	4 (44.4)	5.4 ± 1.6
Ff	39.2 ± 2.6	20.7 ± 4.3	1.3 ± 0.3	869.3 ± 79.6	245 ± 29.1^a^	312.9 ± 27.8	104.6 ± 14.2	5 (62.5)	22 (61.1)	22 (61.1)	5 (55.6)	4.6 ± 1.1
ff	40.2 ± 8.5	24.2 ± 8.1	0.4 ± 0.1	857.3 ± 185.4	139.6 ± 15.6	377.5 ± 74.8	66.3 ± 12.3	0	4 (11.1)	5 (13.9)	0	11.5 ± 8.8
TaqI												
TT	37.8 ± 3.1	11.4 ± 3.1	0.8 ± 0.3	869.3 ± 108.5	229.5 ± 25.3	317.5 ± 36.6	91.9 ± 9.3	3 (37.5)	12 (33.3)	14 (38.9)	2 (33.3)	2.1 ± 0.8
Tt	39.6 ± 2.7	24.3 ± 4.4	1.5 ± 0.3	912.7 ± 74.9	248.2 ± 3	335.9 ± 28.2	110.6 ± 17.8	4 (50)	21 (58.3)	19 (52.8)	6 (66.7)	7.4 ± 1.8
tt	46.5 ± 4.7	8.7 ± 3	1.97 ± 0.7	756.8 ± 141	149.3 ± 22.3	330.6 ± 75	73.3 ± 13.6	1 (12.5)	3 (8.3)	3 (8.3)	0	6.8 ± 3.9

Data are shown as mean ± standard error. IGF1 (%ULN): IGF1/upper limit of normal (age-matched) ∗ 100.

^a^Ff versus ff *P* = 0.0049 (Mann-Whitney *U* test).
